# Attitude of Self-Medication Among Pharmacy Students in King Abdulaziz University, Jeddah, Saudi Arabia: A Cross-Sectional Survey

**DOI:** 10.7759/cureus.33634

**Published:** 2023-01-11

**Authors:** Abdulhamid Althagafi, Nabil Alhakamy, Ahmed Al-Ghamdi, Wesam ShaikhOmar, Fuad Bukhari, Abdulrahman Jowharji, Abduljawad Alsulami, Deem Al-Blaihed, Shimaa T Ibrahim

**Affiliations:** 1 Pharmacy, King Abdulaziz University, Jeddah, SAU; 2 Pharmaceutics, King Abdulaziz University, Jeddah, SAU; 3 Center of Research Excellence for Drug Research and Pharmaceutical Industries, King Abdulaziz University, Jeddah, SAU; 4 Mohamed Saeed Tamer Chair for Pharmaceutical Industries, King Abdulaziz University, Jeddah, SAU; 5 Pediatric Dentistry, King Abdulaziz University Faculty of Dentistry, Jeddah, SAU

**Keywords:** cross sectional studies, attitude, pharmacy, students, ‘self-medication’

## Abstract

Background/purpose

Self-medication is a public health concern because of the potential for medication overuse or abuse, as well as the physical, social, and psychological consequences. In Saudi Arabia, self-medication is common, especially among health science students. Inappropriate self-medication can cause several adverse effects, such as increasing the risk of medication abuse or delaying hospital appointments due to concealing specific symptoms with some medications. Therefore, our study aims to investigate and evaluate health science students' practices, awareness, and attitudes towards self-medication in the Faculty of Pharmacy at King Abdulaziz University, Jeddah, Saudi Arabia.

Materials and methods

A cross-sectional study was conducted using an online self-administered survey to measure the attitude, awareness, and prevalence of self-medication among pharmacy students at King Abdulaziz University in Jeddah, Saudi Arabia. Students in the pharmacy program from the first to the sixth year were invited to participate in the study from April 2019 to June 2019. Raosoft was used to compute the sample size (*n* = 235) with a 5% margin of error and a 95% confidence range.

Results

The factors associated with significant effects were an academic year (*p* = 0.001), smoking (*p* = 0.018), average sleeping time (*p* = 0.032), having any headache (*p* = 0.022), and their opinion about self-medication (*p *< 0.0001).

Conclusion

According to the study, the self-medication of analgesics is common among pharmacy students, and the most used medication was paracetamol.

## Introduction

Self-medication is a global concern and focal issue that has received attention in healthcare systems internationally [1]. The World Health Organization (WHO) describes self-medication as, "the selection and use of medicines by individuals to treat self-recognized illnesses or symptoms" [2]. The increased accessibility of medications and online websites that provide methods of self-diagnosis resulted in significant awareness of health issues, which consequently associated with the involvement of direct self-medication without medical professional consultation [3].

Self-medication practices can be viewed as an alternative method to overcome expensive and time-consuming clinical visits to health care professionals or emergency department visits. This behavior could be attributed to individuals' beliefs that their symptoms are not severe and do not require medical attention, and the convenience of obtaining medication from a community pharmacy without a prescription [4-6]. In addition, increased over-the-counter (OTC) medication options for symptoms such as flu-like symptoms, that is, headache, cold, muscle soreness, and general body aches, might be associated with the observed growth in self-medication prevalence [7]. Among the most common medications used for self-medication, pain management medications such as paracetamol and nonsteroidal anti-inflammatory drugs (NSAIDs) were widely used. The prevalence of self-medication was high in many countries and reached 7% in some countries [8]. Moreover, self-medication has a financial aspect that cannot be neglected. In 2018, the cost of self-medication was estimated to be $34.1 billion compared to $16.8 billion in 2008, with analgesics' estimated sales of $4.3 billion in 2018. The increased healthcare expenses, such as physicians' office visits, longer waiting times in physician clinics or ER visits, and expensive insurance costs, can explain this increase in cost [9].

However, self-medication practices carry certain complications, such as administering inappropriate medication; delaying in seeking appropriate medical attention, and subsequently, delaying appropriate medication management; the occurrence of potential adverse reactions; the occurrence of harmful drug interaction; the masking of underlying diseases; and increased risk of dependence and abuse [10].

Responsible self-medication entails using approved medications that are available without a prescription and are considered relatively safe and effective when taken as recommended [11]. The term "responsible self-medication" was coined to emphasize the potential individual and social benefits of self-medication practices, such as increased access to effective treatment, reduced pressure on the health care system due to fewer physician visits, increased patient autonomy, and lowered costs to third-party payers such as the government or insurance companies [12]. Furthermore, the WHO recognizes the importance of self-medication in the health care system, and acknowledges that responsible self-medication requires adequate support of medicinal products with all necessary information, such as a patient information leaflet, as well as an increase in public's general knowledge, education, and socioeconomic status [13].

In Saudi Arabia, self-medication is common, especially among health science students. Self-monitoring has not been investigated among pharmacy students at Saudi Arabia's King Abdulaziz University in Jeddah. This study aimed to examine and assess students' awareness and attitudes toward self-medication at King Abdulaziz University's Faculty of Pharmacy in Jeddah, Saudi Arabia. To the best of our knowledge, this is the first research on self-medication behaviours among pharmacy students in Jeddah.

## Materials and methods

Study design

This is a cross-sectional study that was conducted to evaluate self-medication behaviours among pharmacy students in Jeddah. This study used the Strengthening the Reporting of Observational Studies in Epidemiology (STROBE) criteria [14].

Ethical approval

The Research Ethics Committee authorized this study from the Faculty of Pharmacy at King Abdulaziz University in Jeddah, Saudi Arabia. The institutional review board was granted for this study (269-19) on 2nd April 2019.

Participants

There are approximately 600 students in King Abdulaziz University's Doctor of Pharmacy (PharmD) program in Jeddah, Saudi Arabia. This is a direct PharmD program for six years. The questionnaire was distributed to all students from the second to the last year between April and June 2019.

Sample size

Based on the Raosoft online sample size calculator with a 5% margin of error and 95% confidence interval, the calculated sample size was 237 students based on the previous study performed in Saudi Arabia among university students - number of students was about 600-650 with a response rate 91% [15]. Therefore, Raosoft was used to compute the sample size (n = 235) with a 5% margin of error and a 95% confidence range [16]. Data collection was halted when 80% of the questionnaire surveys were completed.

Study tool

A predesigned, validated, and structured questionnaire was used to assess pharmacy students' prevalence, attitude, and awareness of self-medication [15, 17]. The survey was distributed to each student through an email system connected to the college, and students received a weekly reminder to complete and submit the survey.

The questionnaire was made available to participants in English via the university's communication center via the student's email system. At the beginning of the questionnaire, participants received a consent form that explained the study's objective and confirmed their identification and confidentiality. Participants had the option to accept or decline participation. Declined participants were excluded from the study.

The questionnaire was composed of 30 questions that included the following data: gender (male or female), age, academic year, nationality, exercise level, smoking, average sleeping time, average daily study time, doctor visits, history of diseases, history of medications, headache type, opinion and reasons on OTC self-medication, analgesics consumption, and safety of the analgesic consumption.

Validity and reliability of study tool

A self-administered validated questionnaire, Study Instrument And Data Collection, was adapted from the previous study conducted by Al Essa et al. in 2019 [15]. Three lecturers from the clinical pharmacy department revised the survey to validate the contents. A pilot study did survey reliability by administering the survey to 10 faculties and 20 students and repeating these studies one week later. Cronbach's alpha coefficient was 0.73, indicating that the survey could be used in this study.

Data collection & outcomes

The consent was implied upon completing and submitting the survey. The data was password protected to uphold the confidentiality of the data and the participants' privacy, and the data were only utilized for research reasons. The primary outcomes were the prevalence of OTC analgesic use. Secondary outcomes included an indication, motivation, knowledge, and awareness of self-medication safety. Data extraction was carried out using a password-protected excel sheet by trained pharmacy interns. Data were reviewed for inconsistency, logical ranges, and missing variables. All data were collected anonymously, and data collectors were blinded throughout the study.

Data analysis

Online responses were subject to tests for completeness and consistency before analysis. Incomplete or repeated responses were not included in the study. Frequency, percentage, and standard deviation were used to summarize the descriptive data. Microsoft Excel® (Microsoft® Corp., Redmond, WA, USA) was used for data entry, and the Statistical Package for the Social Sciences version 21 (IBM Corp., Armonk, NY, USA) for data analysis. Measures of central tendencies and all statistical test results were considered significant if the p-value was ≤ 0.05.

## Results

A total of 238 responses were received, with a response rate of 39.6%. Regarding demographic characteristics, the mean age was (21.97 ± 1.170), with the majority of respondents being male (58.4%). The rest of the demographic data is shown in Table 1. Of the 238 participants, 119 (50%) reported an average sleeping time between 7-9 hours, 185 (77.7%) reported an average estimated study time between 1-3 hours, 209 (87.8%) visited the doctor one to three times in the previous year, 201 (84.5%) had no known illnesses, 164 (68.9%) were not actively taking any prescribed medications, 183 (76.9%) did not have any type of headache, and 113 (47.5%) thought that self-medication was acceptable only after receiving advice from doctor/pharmacist. Finally, 181 (76.9%) admitted to self-medication without referring physicians and pharmacists (Table 1).

**Table 1:** Demographic Information

Characteristics		N = 238, (%)
Gender		
Male		139 (58.4)
Female		99 (41.6)
Students' academic years		
2^nd^ year		55 (23.1)
3^rd^ year		45 (18.9)
4^th^ year		40 (16.8)
5^th^ year		50 (21.0)
Intern		45 (20.2)
Nationality		
Saudi	233 (97.9)	
Non-Saudi	5 (2.1)	
Occasional exercise		
Yes	79 (33.2)	
No	159 (66.8)	
Smoking		
Yes	40 (16.8)	
No	198 (83.2)	
Average sleeping time (hours) n (%)		
<7	114 (47.9)	
7–9	119 (50)	
>9	5 (2.1)	
Daily study time (hours) n (%)		
1–3	185 (77)	
4–6	45 (18.9)	
7–10	8 (3.4)	
Visits to doctor (previous year) n (%)		
1–3	209 (87.8)	
4–6	25 (10.5)	
More than 6	4 (1.7)	
Any past medical history (%)		
Yes	37 (15.5)	
No	201 (84.5)	
Past medical history		
None	199 (83.6)	
Asthma	8 (3.4)	
Anxiety	1 (0.4)	
Depression	1 (0.4)	
Diabetes	1 (0.4)	
Hypertension	2 (0.8)	
Rhinitis	4 (1.7)	
Other allergies	1 (0.4)	
Other	9 (3.8)	
Medication prescribed by physician, n (%)		
Yes	74 (31.3)	
No	164 (68.9)	
Headache n (%)		
Yes	55 (23.1)	
No	183 (76.9)	
Type of headache n (%)		
Tension related	19 (8)	
Migraine	7 (2.9)	
Chronic	1 (0.4)	
Sinus-related	8 (3.4)	
Other	9 (3.8)	
Missing	1 (0.4)	
Opinion about self-medication, n (%)		
OK, only after receiving advice from a doctor/pharmacist	113 (47.5)	
OK, for some problems without consulting a doctor/pharmacist	94 (39.5)	
Always unjust, if not examined by a doctor	17 (7.1)	
The first method of treatment for all problems	14 (5.9)	
Practiced self-medication in past year		
Yes	183 (76.9)	
No	55 (23.1)	

Of the participants, 202 (84.9%) had used OTC analgesics in the past year. Paracetamol was the most common OTC medication used (55%) for headache treatment. According to participants, the majority (67%) believed they had adequate knowledge regarding the side effect profile of OTC medications, and 79.8% of participants did not experience any side effects. Regarding the frequency of purchasing OTC medication, 58% of participants stated that they purchase OTC medication regularly every month (Table 2).

**Table 2:** Analgesics Consumption (Primary Outcome)

Question	N = 238 (%)
Have you used analgesics in the past year?	
Yes	202 (84.9)
No	36 (15.1)
What type of analgesics have you used?	
Paracetamol	131 (55)
NSAIDs	8 (3.4)
Opioids	1 (0.4)
Other	4 (1.7)
What was the indication for analgesics consumption?	
Headache	48 (20.2)
Fever	5 (2.1)
Muscle pain	3 (1.3)
Stomach pain	1 (0.4)
Menstrual cramps	9 (3.8)
Other	8 (3.4)
What was the frequency of analgesics use?	
Valid	25 (10.5)
Almost daily	22 (9.2)
Monthly	138 (58)
Yearly	38 (16)
Other	15 (6.3)
Were you satisfied with analgesics use?	
Valid	25 (10.5)
Yes	194 (81.5)
No	19 (8)
Have you experienced any side effects from analgesics use?	
Valid	23 (9.7)
Yes	25 (10.5)
No	190 (79.8)
Are you aware that analgesics have side effects?	
Yes	161 (67.6)
No	77 (32.4)

Some participants (27%) believed they had adequate knowledge about self-medication. Self-medication was linked to some participants (34.5%) believing they wanted to take an active role in their health. Another explanation for self-medication was the long waiting time in the physician's office considering that over half of participants (51.7%) attributed their self-medication practices for this reason. Respondents think using a high dose of OTC analgesics can lead to adverse effects (Table 3).

**Table 3:** Reasons for Self-Medication and Safety of Analgesics Consumption (Likert Items)

Statement	N = 238 (%)
I have adequate knowledge of medication and disease	
Strongly disagree	16 (6.7)
Disagree	25 (10.5)
Neutral	44 (18.5)
Agree	62 (26.1)
Strongly Agree	36 (15.1)
I want to play an active role in my health	
Strongly disagree	7 (2.9)
Disagree	9 (3.8)
Neutral	34 (14.3)
Agree	51 (21.4)
Strongly Agree	82 (34.5)
I do not want to visit a physician for a long waiting time	
Strongly disagree	12 (5)
Disagree	19 (8)
Neutral	29 (12.2)
Agree	51 (21.4)
Strongly Agree	72 (30.3)
Simultaneous use of analgesics with other medications can be dangerous	
Strongly disagree	9 (3.8)
Disagree	35 (14.7)
Neutral	70 (29.4)
Agree	59 (24.8)
Strongly agree	65 (27.3)
Increasing doses of analgesic medications can be dangerous	
Strongly disagree	4 (1.7)
Disagree	4 (1.7)
Neutral	21 (8.8)
Agree	59 (24.8)
Strongly agree	150 (63)

## Discussion

This survey aimed to assess knowledge and perception of self-medication among pharmacy students at King Abdulaziz University's Faculty of Pharmacy. According to our study, the prevalence of self-medication among the students was relatively high with 76.9% of participants stating they rely on themselves to treat symptoms. This attitude was observed in a similar study that reported a prevalence of self-medication of 73.2% among students in Riyadh that was lower than students of Abha, Saudi Arabia (98%) [17]. In addition, our findings were consistent with those of students from Jordan and the United Arab Emirates where self-medication prevalence reached 96% and 86%, respectively [18, 19]. Our study contrasted with a previous survey study that showed a comparable decrease in self-medication prevalence among pharmacy students (29.6%) [20]. Students' minimal interest and tendency to wait for long times at physicians' offices and an increased desire to play an active role in their own health can explain this observation.

The overall adequacy of knowledge and attitudes about self-medication among the students in this study were reasonable. In addition, the majority of participants (67%) thought they had appropriate knowledge about the adverse effects of most OTC medications, and had not experienced any side effects. Analgesics were the most commonly reported medication used in previous studies [19], which is consistent with our finding in that the majority of our participants (84.9%) stated they consumed OTC analgesics.

Our study showed a minimal association between age, gender, and prevalence of self-medication. Participants stated they relied on self-medication regardless of age or academic year, and no differences in the prevalence of self-medication were noticed. This is in contrast with other studies that found age groups significantly affected the self-medication behaviors. These results are similar to the results of a survey study conducted in King Khalid University, where the prevalence of students who used self-medication to treat non-serious illnesses and minor symptoms was also not related to age, gender, or academic year [21]. In addition, our study showed that 113 students (47.5%) thought self-medication was acceptable only after receiving a consultation from a healthcare professional, such as from pharmacists at community pharmacies.

The WHO considers self-medication as a self-care method to decrease the burden on healthcare professionals; it also encourages regulatory bodies to monitor continuously pharmaceutical products available to the public [13]. Self-medication can only be justified in the competent hands of people who are aware of the medication's nature and can identify the drug's side effects. The increased manufacturing of OTC medications and the wide variety and availability of these medications allow individuals to have multiple options in managing their symptoms [22]. On the other hand, increasing the list of OTC pharmaceuticals is controversial, especially in less-educated societies with poor health systems where patients bear the most medical care costs. Furthermore, it is reasonable that a greater prevalence of self-medication is associated with less control and easy access to medicine [23]. Furthermore, consistent with our findings, one study reported that headaches, fever, general body aches, and other flu-like symptoms were the most complaints individuals relied on themselves to treat [15].

Our study is not without limitations. The self-reporting nature of the study places it in a risk of recall bias since participants heavily relied on their memory to answer the questions distributed to them. In addition, the generalizability of the study is subject to questioning since this study was including students from one pharmacy school, however, similar results were noticed in other previous studies.

More studies are needed to assess the prevalence of self-medication among the health sciences students in different cities and to investigate the numerous factors that influence self-medication, such as student awareness and knowledge of the benefits and drawbacks. Because pharmacy students will be future healthcare professionals who will make significant contributions to the public healthcare system, their perspectives must be recognized and they must be appropriately trained to practice logical and responsible self-medication.

## Conclusions

This study shows a high prevalence of self-medication of analgesic use among pharmacy students. The most prescribed pharmaceutical was paracetamol (analgesic), which is used to treat headaches and cramps. Student knowledge was sufficient regarding analgesic safety. Roughly three-quarters of the students practiced self-medication in the previous year. Apart from those pharmaceuticals that are safe to use in the general population, educating students about the side effects is necessary.

## Appendices

**Table 4:** The Validated English Questionnaire

Demographic	Male
	Female
Academic year	2^nd^ year
	3^rd^ year
	4^th^ year
	5^th^ year
	Inter
Age (Please type your age)	
Nationality	Saudi
	Non-Saudi
Occasional exercise	Yes
	No
Smoking	Yes
	No
Types of smoking	Cigarettes
	Shisha
	Others
What is your average sleeping time?	< 7 hrs
	7–9 hrs
	More than 9 hrs
What is your average daily study time?	1–3 hrs
	4–6 hrs
	7–10 hrs
How many times have you visited a doctor in the previous year?	1–3 times
	4–6 times
	More than 6
Do you have any diseases?	Yes
	No
What type of diseases do you have? (You can choose up to 4)	Hypertension
	Skin disease
	Epilepsy
	Anxiety
	Psychiatric disorder
	Cancer
	Diabetes
	Asthma
	Kidney disease
	Depression
	Rhinitis
	Other allergies
	None
Any medication prescribed by the physician?	Yes
	No
Do you have any kind of headache?	Yes
	No
What are the types of headaches you have (if you choose yes to the previous question)	Migraine
	Tension related
	Sinus related
	Chronic
	Other
	Missing
	None
What's your opinion about self-medication?	Always unjust, if not examined by the doctor
	Okay only after receiving advice from a doctor/pharmacist
	Ok for some problems without consulting a doctor
	First method of treatment for all problems
Have you practiced self-medication in the past year?	Yes
	No

**Table 5:** Reasons for self-medication only if answered yes to the previous question

I have adequate knowledge of medication	Strongly disagree 1	Disagree 2	Neutral 3	Agree 4	Strongly agree 5
I don't want to burden physicians; my illness is not severe	Strongly disagree 1	Disagree 2	Neutral 3	Agree 4	Strongly agree 5
I want to play an active role in my health	Strongly disagree 1	Disagree 2	Neutral 3	Agree 4	Strongly agree 5
I do not want to visit a physician because of the long waiting time	Strongly disagree 1	Disagree 2	Neutral 3	Agree 4	Strongly agree 5

**Table 6:** Analgesics Consumption

Have you used analgesics in the past year?	
Yes	No
What type of analgesics that you use?	
Paracetamol, NSAIDs, Opioids, Others	
What was the indication for its consumption? (Choose up to 4)	
Headache, Fever, Cold, Muscle pain, Stomach pain, Menstrual cramps, Allergy, Other	
What is the frequency of analgesic use?	
Almost daily, Monthly, Yearly, Other	
Were you satisfied with analgesics use?	
Yes	No
Have you experienced any side effects from analgesic use?	
Yes	No
Are you aware that analgesics have side effects?	
Yes	No

**Table 7:** Safety of Analgesics Consumption

Simultaneous use of analgesics with other drugs can be dangerous.				
Strongly disagree 1	Disagree 2	Neutral 3	Agree 4	Strongly agree 5
Increasing the dosage of analgesic drugs can be dangerous.				
Strongly disagree 1	Disagree 2	Neutral 3	Agree 4	Strongly agree 5
